# Ammonium [(1*S*)-(*endo*,*anti*)]-(−)-3-bromo­camphor-8-sulfonate

**DOI:** 10.1107/S1600536810022804

**Published:** 2010-06-18

**Authors:** Muhammad Athar Abbasi, Aziz-ur-Rehman, Mehmet Akkurt, Muhammad Jahangir, Seik Weng Ng, Islam Ullah Khan

**Affiliations:** a Department of Chemistry, Government College University, Lahore 54000, Pakistan; b Department of Physics, Faculty of Arts and Sciences, Erciyes University, 38039 Kayseri, Turkey; c Department of Chemistry, University of Malaya, 50603 Kuala Lumpur, Malaysia

## Abstract

In the title mol­ecular salt, NH_4_^+^·C_10_H_14_BrO_4_S^−^, the norbornane skeleton of the anion is composed of two five-membered rings in envelope conformations and a six-membered ring with one Br atom, one carbonyl O atom and a methyl group held in a boat conformation by a bridging methyl­ene group. Short intra­molecular C—H⋯O and C—H⋯Br inter­actions occur. In the crystal, the component ions are linked by inter­molecular N—H⋯O and C—H⋯O hydrogen bonds.

## Related literature

For further synthetic details, see: Smith *et al.* (2008 ▶). For other structures with the norbornane skeleton, see: Jauch *et al.* (1992 ▶); Ustabaş *et al.* (2006 ▶); Ersanlı *et al.* (2005 ▶). For the use of 3-bromo­camphor-8-sulfonic acid and its ammonium salts as chiral auxillaries for the optical resolution of enanti­omeric amines through diasteriomeric salt formation, see: Bálint *et al.* (1999 ▶); Pellati *et al.* (2010 ▶); Roy *et al.* (2009 ▶); Zhao *et al.* (2002 ▶). For puckering parameters, see: Cremer & Pople (1975 ▶).
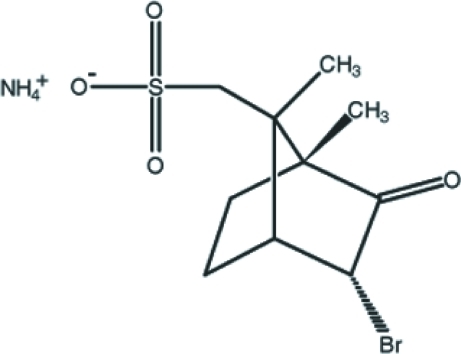


## Experimental

### 

#### Crystal data

NH_4_^+^·C_10_H_14_BrO_4_S^−^
*M* *_r_* = 328.22
Monoclinic, 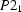

*a* = 7.2449 (2) Å
*b* = 7.0049 (1) Å
*c* = 13.2428 (3) Å
β = 104.704 (1)°
*V* = 650.06 (3) Å^3^
*Z* = 2
Mo *K*α radiation
μ = 3.33 mm^−1^
*T* = 296 K
0.42 × 0.14 × 0.11 mm

#### Data collection

Bruker Kappa APEXII CCD diffractometer
Absorption correction: refined from Δ*F* (*XABS2*; Parkin *et al.*, 1995 ▶) *T*_min_ = 0.336, *T*_max_ = 0.711
2775 measured reflections
2775 independent reflections
2586 reflections with *I* > 2σ(*I*)

#### Refinement

*R*[*F* ^2^ > 2σ(*F* ^2^)] = 0.027
*wR*(*F* ^2^) = 0.065
*S* = 1.03
2775 reflections
168 parameters
5 restraints
H atoms treated by a mixture of independent and constrained refinement
Δρ_max_ = 0.35 e Å^−3^
Δρ_min_ = −0.47 e Å^−3^
Absolute structure: Flack (1983 ▶), 1155 Freidel pairs
Flack parameter: −0.021 (7)

### 

Data collection: *APEX2* (Bruker, 2007 ▶); cell refinement: *SAINT* (Bruker, 2007 ▶); data reduction: *SAINT*; program(s) used to solve structure: *SHELXS97* (Sheldrick, 2008 ▶); program(s) used to refine structure: *SHELXL97* (Sheldrick, 2008 ▶); molecular graphics: *ORTEP-3* (Farrugia, 1997 ▶); software used to prepare material for publication: *WinGX* (Farrugia, 1999 ▶), *PARST* (Nardelli, 1983 ▶) and *PLATON* (Spek, 2009 ▶).

## Supplementary Material

Crystal structure: contains datablocks global, I. DOI: 10.1107/S1600536810022804/hb5484sup1.cif

Structure factors: contains datablocks I. DOI: 10.1107/S1600536810022804/hb5484Isup2.hkl

Additional supplementary materials:  crystallographic information; 3D view; checkCIF report

## Figures and Tables

**Table 1:** Hydrogen-bond geometry (Å, °)

*D*—H⋯*A*	*D*—H	H⋯*A*	*D*⋯*A*	*D*—H⋯*A*
N1—H1*N*⋯O1^i^	0.92 (3)	1.92 (3)	2.835 (4)	173 (3)
N1—H2*N*⋯O2^ii^	0.90 (3)	2.05 (3)	2.899 (3)	157 (3)
N1—H3*N*⋯O2	0.92 (3)	1.97 (3)	2.887 (3)	176 (3)
N1—H4*N*⋯O3^iii^	0.92 (3)	1.93 (3)	2.827 (3)	167 (4)
C4—H4*B*⋯Br1	0.97	2.71	3.221 (3)	113
C8—H8*A*⋯O2	0.96	2.44	3.104 (3)	126
C10—H10⋯O1^i^	0.98	2.49	3.451 (4)	167

Symmetry codes: (i) 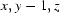; (ii) 
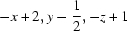
; (iii) 
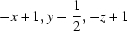
.

**Fig. 1.**
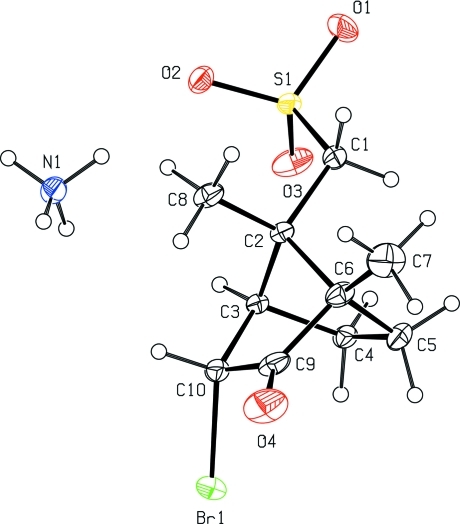
View of (I) with displacement ellipsoids drawn at the 30% probability level.

**Fig. 2.**
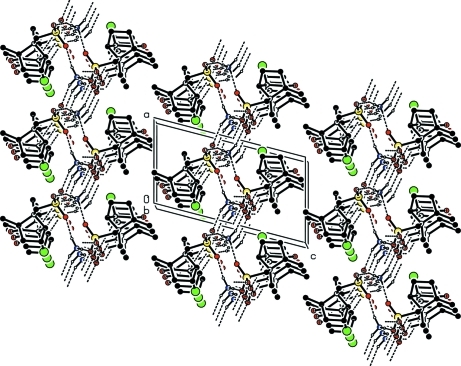
The crystal packing of (I) viewed down the b-axis. The hydrogen-bonds are drawn as a dashed lines. H-atoms not involved in hydrogen bonds have been omitted for clarity.

## supplementary crystallographic information

### Comment

3-Bromocamphor-8-sulfonic acid and its ammonium salts have extensively been used as chiral auxillaries for the optical resolution of a number of enantiomeric amines through diasteriomeric salt formation (Bálint *et al.*, 1999; Zhao *et al.*, 2002; Roy *et al.*, 2009; Pellati *et al.*, 2010).

In the bicyclo[2.2.1]heptane (norbornane) skeleton of the title compound, (I), (Fig. 1), the two five-membered rings have envelope conformations, with atom C2 displaced by 0.365 (3) Å from the C2–C6 plane [the puckering parameters (Cremer & Pople, 1975) are Q_2_ = 0.573 (3) Å and φ_2_ = 5.3 (3)°] and by 0.397 (3) Å from the C2/C3/C6/C9/C10 plane [the puckering parameters: Q_2_ = 0.615 (3) Å and φ_2_ = 181.6 (3)°] and the six-membered ring (C3–C6/C9/C10) adopts a boat conformation by the puckering parameters *Q*_T_ = 0.970 (3) Å, θ = 92.03 (18)° and φ = 357.34 (19) °.

In (I), the C—C single-bond lengths range from 1.491 (5) to 1.575 (4) Å, with a mean value of 1.535 (4) Å. In the bicyclo[2.2.1]heptane fragment, the angles between planes A (C3/C2/C6), B (C3–C6) and C (C3/C6/C9/C10) are as follows: A/B= 53.65 (19)°, A/C= 58.14 (18)° and B/C= 68.22 (13)°.

In the crystal, adjacent molecules of (I) are linked by intermolecular N—H···O and C—H···O hydrogen bonds (Table 1, Fig. 2).

### Experimental

3-Bromocamphor-8-sulfonic acid ammonium salt was prepared by modification in the reported method (Smith *et al.*, 2008). 3-Bromocamphor-8-sulfonic acid (1 g) was dissolved in 15 ml of ethanol and then 6 ml of NH_3_ solution were added. The mixture was stirred until a clear solution was observed (about 20 min). The solution was slowly concentrated on water bath to half the volume over a 2 h period. The concentrate was allowed to crystallize undisturbed for 48 h.  The resulting colourless prisms of (I) were carefully separated by filteration and washed with three 0.5-ml portions of petroleum ether.

### Refinement

In the ammonium ion, H atoms bound to N atoms were located in difference Fourier maps and their positional parameters were refined freely using a *DFIX* instruction [N—H = 0.93 (3) Å] in *SHELXL97*, with *U*_iso_(H) = 1.5*U*_eq_(N). H atoms bound to C atoms were placed in idealized positions and refined using a riding model with C—H = 0.96, 0.97 and 0.98 Å for CH_3_, CH_2_ and CH, respectively. *U*_iso_(H) values were set at 1.5*U*_eq_(C) for the methyl groups, and 1.2*U*_eq_*U*_eq_(C) for other H atoms.

### Crystal data

**Table:**

NH_4_^+^·C_10_H_14_BrO_4_S^−^	*F*(000) = 336
*M**_r_* = 328.22	*D*_x_ = 1.677 Mg m^−^^3^
Monoclinic, *P*2_1_	Mo *K*α radiation, λ = 0.71073 Å
Hall symbol: P 2yb	Cell parameters from 3356 reflections
*a* = 7.2449 (2) Å	θ = 2.9–28.3°
*b* = 7.0049 (1) Å	µ = 3.33 mm^−^^1^
*c* = 13.2428 (3) Å	*T* = 296 K
β = 104.704 (1)°	Prism, colourless
*V* = 650.06 (3) Å^3^	0.42 × 0.14 × 0.11 mm
*Z* = 2	

### Data collection

**Table:**

Bruker Kappa APEXII CCD diffractometer	2775 independent reflections
Radiation source: sealed tube	2586 reflections with *I* > 2σ(*I*)
graphite	*R*_int_ = 0.0000
φ and ω scans	θ_max_ = 27.5°, θ_min_ = 3.3°
Absorption correction: part of the refinement model (Δ*F*) (*XABS2*; Parkin *et al.*, 1995; quadratic fit to sin(θ)/λ - 18 parameters)	*h* = −9→9
*T*_min_ = 0.336, *T*_max_ = 0.711	*k* = −8→9
2775 measured reflections	*l* = 0→17

### Refinement

**Table:**

Refinement on *F*^2^	Secondary atom site location: difference Fourier map
Least-squares matrix: full	Hydrogen site location: inferred from neighbouring sites
*R*[*F*^2^ > 2σ(*F*^2^)] = 0.027	H atoms treated by a mixture of independent and constrained refinement
*wR*(*F*^2^) = 0.065	*w* = 1/[σ^2^(*F*_o_^2^) + (0.033*P*)^2^ + 0.1814*P*] where *P* = (*F*_o_^2^ + 2*F*_c_^2^)/3
*S* = 1.03	(Δ/σ)_max_ < 0.001
2775 reflections	Δρ_max_ = 0.35 e Å^−^^3^
168 parameters	Δρ_min_ = −0.47 e Å^−^^3^
5 restraints	Absolute structure: Flack (1983), 1155 Freidel pairs
Primary atom site location: structure-invariant direct methods	Flack parameter: −0.021 (7)

### Special details

**Table:**

Geometry. Bond distances, angles *etc*. have been calculated using the rounded fractional coordinates. All su's are estimated from the variances of the (full) variance-covariance matrix. The cell e.s.d.'s are taken into account in the estimation of distances, angles and torsion angles
Refinement. Refinement on *F*^2^ for ALL reflections except those flagged by the user for potential systematic errors. Weighted *R*-factors *wR* and all goodnesses of fit *S* are based on *F*^2^, conventional *R*-factors *R* are based on *F*, with *F* set to zero for negative *F*^2^. The observed criterion of *F*^2^ > σ(*F*^2^) is used only for calculating -*R*-factor-obs *etc*. and is not relevant to the choice of reflections for refinement. *R*-factors based on *F*^2^ are statistically about twice as large as those based on *F*, and *R*-factors based on ALL data will be even larger.

### Fractional atomic coordinates and isotropic or equivalent isotropic displacement parameters (Å^2^)

**Table:**

	*x*	*y*	*z*	*U*_iso_*/*U*_eq_	
Br1	0.09450 (4)	0.00017 (4)	0.27520 (3)	0.0440 (1)	
S1	0.65799 (9)	0.68509 (9)	0.36429 (5)	0.0242 (2)	
O1	0.7216 (3)	0.8793 (3)	0.3541 (2)	0.0434 (8)	
O2	0.8199 (3)	0.5560 (3)	0.39714 (16)	0.0332 (6)	
O3	0.5283 (3)	0.6697 (4)	0.42972 (17)	0.0448 (8)	
O4	0.2268 (4)	0.0517 (4)	0.0568 (2)	0.0596 (10)	
C1	0.5311 (4)	0.6201 (4)	0.2339 (2)	0.0277 (8)	
C2	0.4754 (4)	0.4086 (4)	0.2154 (2)	0.0213 (7)	
C3	0.3597 (3)	0.3263 (4)	0.2899 (2)	0.0223 (7)	
C4	0.1802 (4)	0.4500 (4)	0.2647 (2)	0.0272 (8)	
C5	0.1351 (4)	0.4758 (5)	0.1462 (2)	0.0352 (9)	
C6	0.3067 (4)	0.3804 (4)	0.1148 (2)	0.0310 (9)	
C7	0.3318 (6)	0.4411 (6)	0.0112 (2)	0.0519 (13)	
C8	0.6514 (4)	0.2949 (4)	0.2067 (2)	0.0316 (9)	
C9	0.2719 (4)	0.1690 (5)	0.1248 (2)	0.0342 (9)	
C10	0.3097 (4)	0.1283 (4)	0.2418 (2)	0.0298 (8)	
N1	0.8032 (3)	0.1906 (4)	0.4952 (2)	0.0311 (7)	
H1A	0.41550	0.69600	0.21450	0.0330*	
H1B	0.60920	0.65480	0.18710	0.0330*	
H3	0.42880	0.32500	0.36380	0.0270*	
H4A	0.20390	0.57210	0.30010	0.0330*	
H4B	0.07600	0.38650	0.28480	0.0330*	
H5A	0.12590	0.61000	0.12770	0.0420*	
H5B	0.01620	0.41320	0.11210	0.0420*	
H7A	0.22080	0.40620	−0.04250	0.0780*	
H7B	0.44200	0.37920	−0.00150	0.0780*	
H7C	0.34880	0.57700	0.01080	0.0780*	
H8A	0.74270	0.29220	0.27340	0.0470*	
H8B	0.70750	0.35440	0.15630	0.0470*	
H8C	0.61420	0.16680	0.18500	0.0470*	
H10	0.42240	0.04610	0.26290	0.0360*	
H1N	0.775 (5)	0.096 (4)	0.445 (2)	0.0470*	
H2N	0.914 (4)	0.170 (6)	0.543 (2)	0.0470*	
H3N	0.806 (5)	0.305 (4)	0.461 (3)	0.0470*	
H4N	0.706 (4)	0.195 (6)	0.528 (3)	0.0470*	

### Atomic displacement parameters (Å^2^)

**Table:**

	*U*^11^	*U*^22^	*U*^33^	*U*^12^	*U*^13^	*U*^23^
Br1	0.0329 (2)	0.0300 (2)	0.0673 (2)	−0.0085 (1)	0.0093 (1)	0.0087 (2)
S1	0.0199 (3)	0.0209 (3)	0.0303 (3)	−0.0028 (2)	0.0036 (2)	−0.0024 (3)
O1	0.0492 (13)	0.0217 (11)	0.0526 (15)	−0.0096 (9)	0.0007 (11)	−0.0030 (9)
O2	0.0246 (9)	0.0298 (11)	0.0393 (12)	0.0024 (7)	−0.0029 (8)	−0.0036 (8)
O3	0.0304 (10)	0.0674 (17)	0.0391 (13)	−0.0101 (11)	0.0133 (9)	−0.0175 (12)
O4	0.0552 (15)	0.060 (2)	0.0580 (16)	−0.0138 (12)	0.0042 (12)	−0.0336 (13)
C1	0.0241 (13)	0.0248 (14)	0.0298 (15)	−0.0018 (11)	−0.0010 (11)	0.0010 (11)
C2	0.0168 (12)	0.0239 (13)	0.0226 (13)	−0.0016 (10)	0.0041 (10)	−0.0026 (10)
C3	0.0170 (11)	0.0207 (12)	0.0274 (14)	−0.0020 (9)	0.0022 (10)	−0.0004 (10)
C4	0.0184 (11)	0.0235 (15)	0.0403 (16)	0.0017 (9)	0.0084 (10)	−0.0020 (11)
C5	0.0222 (12)	0.0368 (19)	0.0406 (16)	0.0024 (13)	−0.0030 (11)	0.0017 (14)
C6	0.0239 (13)	0.0409 (17)	0.0248 (15)	−0.0040 (12)	−0.0002 (11)	−0.0024 (12)
C7	0.056 (2)	0.070 (3)	0.0247 (17)	−0.0134 (18)	0.0011 (15)	0.0033 (15)
C8	0.0206 (13)	0.0359 (16)	0.0382 (17)	−0.0007 (12)	0.0074 (11)	−0.0104 (13)
C9	0.0198 (12)	0.0392 (17)	0.0405 (16)	−0.0051 (12)	0.0018 (11)	−0.0130 (14)
C10	0.0213 (12)	0.0212 (13)	0.0444 (17)	−0.0006 (10)	0.0039 (11)	−0.0016 (11)
N1	0.0266 (12)	0.0334 (13)	0.0329 (13)	0.0019 (11)	0.0066 (10)	0.0030 (11)

### Geometric parameters (Å, °)

**Table:**

Br1—C10	1.945 (3)	C6—C7	1.491 (4)
S1—O1	1.454 (2)	C6—C9	1.514 (4)
S1—O2	1.457 (2)	C9—C10	1.530 (4)
S1—O3	1.435 (2)	C1—H1B	0.9700
S1—C1	1.797 (3)	C1—H1A	0.9700
O4—C9	1.201 (4)	C3—H3	0.9800
N1—H1N	0.92 (3)	C4—H4B	0.9700
N1—H2N	0.90 (3)	C4—H4A	0.9700
N1—H3N	0.92 (3)	C5—H5A	0.9700
N1—H4N	0.92 (3)	C5—H5B	0.9700
C1—C2	1.539 (4)	C7—H7A	0.9600
C2—C3	1.558 (4)	C7—H7B	0.9600
C2—C6	1.575 (4)	C7—H7C	0.9600
C2—C8	1.532 (4)	C8—H8B	0.9600
C3—C4	1.527 (4)	C8—H8C	0.9600
C3—C10	1.531 (4)	C8—H8A	0.9600
C4—C5	1.530 (4)	C10—H10	0.9800
C5—C6	1.558 (4)		
			
O1—S1—O2	111.00 (13)	C3—C10—C9	102.4 (2)
O1—S1—O3	113.46 (15)	S1—C1—H1B	108.00
O1—S1—C1	104.17 (14)	S1—C1—H1A	108.00
O2—S1—O3	111.96 (14)	C2—C1—H1A	108.00
O2—S1—C1	107.84 (13)	C2—C1—H1B	108.00
O3—S1—C1	107.92 (14)	H1A—C1—H1B	107.00
H3N—N1—H4N	109 (3)	C10—C3—H3	114.00
H2N—N1—H4N	109 (3)	C4—C3—H3	114.00
H1N—N1—H3N	107 (3)	C2—C3—H3	115.00
H1N—N1—H4N	108 (3)	C5—C4—H4A	111.00
H1N—N1—H2N	113 (3)	C3—C4—H4B	111.00
H2N—N1—H3N	111 (3)	C3—C4—H4A	111.00
S1—C1—C2	116.52 (19)	C5—C4—H4B	111.00
C1—C2—C8	108.8 (2)	H4A—C4—H4B	109.00
C1—C2—C6	111.8 (2)	H5A—C5—H5B	109.00
C1—C2—C3	114.6 (2)	C4—C5—H5A	111.00
C6—C2—C8	110.7 (2)	C4—C5—H5B	111.00
C3—C2—C6	93.6 (2)	C6—C5—H5A	111.00
C3—C2—C8	116.6 (2)	C6—C5—H5B	111.00
C4—C3—C10	108.9 (2)	H7B—C7—H7C	109.00
C2—C3—C4	102.5 (2)	C6—C7—H7C	109.00
C2—C3—C10	100.4 (2)	C6—C7—H7A	110.00
C3—C4—C5	103.9 (2)	C6—C7—H7B	109.00
C4—C5—C6	104.2 (2)	H7A—C7—H7B	109.00
C7—C6—C9	114.9 (3)	H7A—C7—H7C	109.00
C2—C6—C7	119.5 (3)	C2—C8—H8B	109.00
C2—C6—C5	102.8 (2)	C2—C8—H8A	109.00
C5—C6—C9	103.6 (2)	H8A—C8—H8C	109.00
C2—C6—C9	99.2 (2)	C2—C8—H8C	109.00
C5—C6—C7	114.5 (3)	H8A—C8—H8B	109.00
O4—C9—C6	128.6 (3)	H8B—C8—H8C	110.00
O4—C9—C10	125.2 (3)	C9—C10—H10	109.00
C6—C9—C10	106.3 (2)	Br1—C10—H10	109.00
Br1—C10—C3	116.24 (18)	C3—C10—H10	109.00
Br1—C10—C9	111.62 (19)		
			
O1—S1—C1—C2	169.7 (2)	C2—C3—C4—C5	39.1 (3)
O2—S1—C1—C2	51.7 (2)	C10—C3—C4—C5	−66.7 (3)
O3—S1—C1—C2	−69.4 (3)	C2—C3—C10—Br1	−159.47 (17)
S1—C1—C2—C3	54.3 (3)	C2—C3—C10—C9	−37.5 (3)
S1—C1—C2—C6	159.2 (2)	C4—C3—C10—Br1	−52.3 (3)
S1—C1—C2—C8	−78.2 (3)	C4—C3—C10—C9	69.7 (3)
C1—C2—C3—C4	61.2 (3)	C3—C4—C5—C6	−5.6 (3)
C1—C2—C3—C10	173.5 (2)	C4—C5—C6—C2	−29.3 (3)
C6—C2—C3—C4	−54.8 (2)	C4—C5—C6—C7	−160.5 (3)
C6—C2—C3—C10	57.5 (2)	C4—C5—C6—C9	73.6 (3)
C8—C2—C3—C4	−170.0 (2)	C2—C6—C9—O4	−144.1 (3)
C8—C2—C3—C10	−57.8 (3)	C2—C6—C9—C10	34.8 (3)
C1—C2—C6—C5	−67.8 (3)	C5—C6—C9—O4	110.3 (4)
C1—C2—C6—C7	60.4 (4)	C5—C6—C9—C10	−70.9 (3)
C1—C2—C6—C9	−174.0 (2)	C7—C6—C9—O4	−15.4 (5)
C3—C2—C6—C5	50.6 (2)	C7—C6—C9—C10	163.5 (3)
C3—C2—C6—C7	178.7 (3)	O4—C9—C10—Br1	−54.7 (4)
C3—C2—C6—C9	−55.7 (2)	O4—C9—C10—C3	−179.8 (3)
C8—C2—C6—C5	170.7 (2)	C6—C9—C10—Br1	126.4 (2)
C8—C2—C6—C7	−61.1 (4)	C6—C9—C10—C3	1.3 (3)
C8—C2—C6—C9	64.5 (3)		

### Hydrogen-bond geometry (Å, °)

**Table:**

*D*—H···*A*	*D*—H	H···*A*	*D*···*A*	*D*—H···*A*
N1—H1N···O1^i^	0.92 (3)	1.92 (3)	2.835 (4)	173 (3)
N1—H2N···O2^ii^	0.90 (3)	2.05 (3)	2.899 (3)	157 (3)
N1—H3N···O2	0.92 (3)	1.97 (3)	2.887 (3)	176 (3)
N1—H4N···O3^iii^	0.92 (3)	1.93 (3)	2.827 (3)	167 (4)
C4—H4B···Br1	0.97	2.71	3.221 (3)	113
C8—H8A···O2	0.96	2.44	3.104 (3)	126
C10—H10···O1^i^	0.98	2.49	3.451 (4)	167

Symmetry codes: (i) *x*, *y*−1, *z*; (ii) −*x*+2, *y*−1/2, −*z*+1; (iii) −*x*+1, *y*−1/2, −*z*+1.
